# Observations on the Powers and Effects of Cold as a Cause of Disease; with Some Remarks on the Best Means of Preventing Its Morbid Effects, &c.

**Published:** 1832-09

**Authors:** John Clendinning

**Affiliations:** Edinburgh and Oxford, Fellow and Censor of the Royal College of Physicians of London, and Physician to the Western Dispensary.


### COLD AS A CAUSE OF DISEASE.

*Observations on the Powers and Effects of Cold as a Cause of Disease; with some Remarks on the best Means of Preventing its Morbid Effects, &c.* By JOHN CLENDINNING, M.D. of Edinburgh and Oxford, Fellow and Censor of the Royal College of Physicians of London, and Physician to the Western Dispensary.

(Concluded from page 32.)

### *Diseases of Cold.*

COLD has a threefold morbific power; as a *physical or chemical* agent, as a *predisposing cause* of disease, and as an *occasional*.

As a physical cause, its operation is definite and simple: it abstracts that caloric whose presence is a necessary condition of functional activity, and has for its effect suspension of the vital action of the organ or organs subjected to its influence. Asphyxia, local or general, and congelation, are the results of extreme refrigeration, and the only disease cold is capable of producing, independently of reaction. In a former place, I have shewn that chilblain, and what has improperly been called local asphyxia, namely, gangrene consequent upon refrigeration, are the results, not simply or directly of abstraction of heat, but of reactive erethism, turgescence, and inflammation.

The mortality from cold as a predisposing cause, is attributable principally to fevers often of a contagious nature; to inflammations of the brain, lungs, heart, liver, and bowels; to hemorrhagic diseases of the brain and lungs; to dropsies of the chest and abdomen; to catarrhal and asthmatic complaints: to scrofulous, icteric, scorbutic, and chlorotic cachexies; to diarrhœas and diabetes; to rheumatic, gouty, and calculous disorders. Of the etiology of several of the diseases above named, so far as connected with the history of morbific cold, I have already, in fact, sufficiently treated in the section on the morbific properties of cold. The diseases I refer to more particularly are asphyxia, and catarrhal and other congeneric affections of the lungs; diarrhœa, dysentery, and other abdominal derangements; scurvy and other humoral cachexies; dropsical and hemorrhagic diseases of the head and other great cavities. Having, therefore, for the better illustration of the morbific agency of cold, already stated in the preceding section, much that would otherwise call for notice here, I shall, under the head of *diseases of cold*, content myself with a rapid enumeration of the effects of that agent in its twofold character, as an occasional and predisposing cause.

In most countries the great mass of mortality is attributable to fevers, and of that class of diseases cold is amongst the most active, predisposing, and indeed also exciting causes. "Quivis ferè morbus epidemicus, vel contagiosus, apud inferiorem populi classem, et exoritur, et quam maximè dominatur, et eradicatur tardius, ita ut levior etiam angustiore loco reclusa, adfectio, *hyberno imprimis tempore, quo cum frigore auræ etiam vitalis allisionem avertere student*, in funestam, mox carcerali nosocomialique; febri, similem ægritudinem terminetur, et in amicos miseriâ exhaustos, predispositos, propagetur. *Quæ substanta corporis nuditate, et refocillantis defectu foci, à summo gelu*, quæ ex habitationibus fuliginosis et impuris, quam fœdi cutis morbi, à neglectâ corporis munditie, et à transpiratione per angustias contineras languente, miserioribus sint proprii, hoc non nisi verbo hic attingo," &c. (J. P. Frank, *de Populorum miseriâ morborum genetrici.*)

"It is needless to mention how much an ample supply of fuel is conducive to health," says a learned experienced writer of this city, "not merely for warmth and for culinary purposes, but as promoting ventilation, which it does, not by the change of air necessarily induced by the current of air up the chimney, but by enabling the poor to admit fresh air in cold weather;" as well, he might have added, as by rendering ablution and cleanliness of every kind practicable and convenient. "It is in the winter season," Sir G. Blane further remarks, "from want of fuel, that typhous infection is most apt to arise, and also to spread." It is obvious that the confinement and inactivity that the indolent and poor voluntarily impose on themselves, for the sake of avoiding the disagreeable sensations of cold weather, must, in various ways, favor the attacks of disease, independently of accumulated effluvia, must predispose to scrofula, chlorosis, amenorrhœa, dropsy, catarrh, pulmonic and other internal inflammations, pectoral, abdominal, and other hemorrhages, &c.

But it is as an exciting or occasional cause that cold is most extensively operative as a morbific agent: there is almost no disease of an acute character, nor any chronic paroxysmal complaint, whose attacks we do not observe produced by it. So extended is its morbific influence as an occasional cause, that even to enumerate the diseases whose explosion it is capable of determining in predisposed subjects, would be almost to repeat the nosological catalogue. Complaints the most different in symptoms, and seat, and result, are amongst its occasional effects: fever of every kind, every variety of inflammation, spasmodic diseases of every kind and degree, from the cramp that annoys the swimmer to the merciless cholera and tetanus. It has been known to occasion every sort of neuralgic disease, every species of vitiated action to which the excretories are liable; it occasions apoplectic, paralytic, epileptic, and hysteric, seizures; and gives rise to every description of indigestion and of asthmatic disease. But of all its powers of noxious action, the most extensively mischievous is probably that by which it occasions internal fluxions, congestions, and inflammations. To this power is owing not only the bulk of the mortality of purely inflammatory diseases, but also a large share of that of continued or essential fevers also. It is now well known that local inflammation is amongst the most frequent and formidable complications of typhoid, eruptive, and other, pyrexiæ: this power I have not been able to include in the list of morbific properties of cold, because it is not by any one property that such local diseases are occasioned; for several distinct and independent organs and elements conspire in the production of the effect; viz. the skin with its nerves, vessels, and blood, and the internal organ with its nerves, blood, and vessels, &c.; and secondly, because the seat and order of the simpler effects or morbid changes, which, beginning with cutaneous refrigeration, terminate in internal visceral disease, are, I conceive, quite uncertain. The explanation usually given of the causation of these diseases is founded on the obscure doctrine of sympathy; the external teguments in the coverings and parenchymata of the viscera are said so to sympathize with each other, that functional disturbance in the former is followed, in susceptible subjects, by similar derangement in one or more of the latter; and, in a practical point of view at least, I readily admit that this doctrine of *sympathy* or *antagonism* is well worthy of consideration; indeed, it has served my distinguished friend Dr. James Johnson for a basis of excellent therapeutical instruction in his very valuable work on the Diseases of India, and other subsequent writings, and furnishes unquestionably many indications of the highest practical utility; but it is still, in a scientific point of view, if I mistake not, very imperfect and unsatisfactory. It furnishes but two links, as it were, of a long chain of causation, and these remote from and unconnected with each other by any bond or force that we can discover. Between the first frigorific impression on the skin of the feet, let us suppose, and the ultimate establishment, at the distance of many hours, perhaps of a day or two, of a fluxion, &c. in the lungs, liver, &c. there is, according to this doctrine, an enormous blank, an interval full of important organic and humoral changes of which sympathy takes no account whatever: under these circumstances, I have considered myself justified in omitting the power of cold now under consideration, in my enumeration of the morbific properties of that agent, leaving open for future inquiry its *modus operandi* as an exciting cause in the origination of visceral disease. Before, however, quitting the subject, I would observe that this question is so entangled with that of predisposition, that, so long as the latter doctrine continues in its present state, a *rudis indigestaque moles* of hypothesis, conjecture, fact, and inference, offering no definite principles or rules of reasoning or action, there is little prospect of its satisfactory solution; and this remark will hold good, I imagine, even after we shall have become very much better acquainted with the intimate nature of several diseases occasioned by cold, of the proximate causes of which we are at present, to a great extent, or for the most part, ignorant.

The morbic influence of cold as an exciting cause is, therefore, almost coextensive with morbific action; nor shall I probably be considered to over-rate the morbific power of cold by any who will take into account, on the one hand, our constant immersion in an atmosphere of very variable temperature and humidity, over whose fluctuations we have little control, and with which it is necessary to our existence that we should retain in strict contact one surface at least of our bodies; a surface whose extent has been estimated at nearly a score of square feet. This estimate of the morbific power of cold is rendered further probable by the reflection that of the three great classes of exciting causes of disease, namely, the moral, the internal or organic, and the external or physical, which last includes atmospherical fluctuations, the third class or external causes are not only the farthest beyond our reach, when quiescent, but the least under our control when in action. In the list of diseases above enumerated as producible by cold, it will have been observed that many, if not all, are common to the two classes; namely, to the class of disorders which it excites as well as that to which it merely predisposes. The coincidence and almost identity between those classes is founded on the nature of things, and depends on a law that pervades the whole domain of pathological etiology. Cold, like almost all other causes of disease, varies in its action according to its own energy and according to the amount of susceptibility in the subject to which it is applied; when intense or aided by a high predisposition, it determines at once the attack of disease; if moderate or not favored by adequate susceptibility, it of itself produces no change, and is null, or merely contributes to complete, in cooperation with other causes, a growing predisposition to some disease which may be called into active operation by perhaps a fresh exposure to cold, and will in every case require the aid of some determining application, change, event, or, in other words, occasional cause. And there is another view of the subject of predisposition, perhaps still more difficult and embarrassing: how it is, in differently predisposed individuals at one time, or in the same individual at different times, refrigeration is followed by such various results; why does cold produce in one person or at one time catarrh, at another pneumonia, or pleurisy, or enteritis, or diarrhœa, or herpes, or erysipelas, or some other one of the thousand febrile inflammatory, nervous, &c. diseases of cold? To this question, which has been already glanced at, and which goes deeper into medical philosophy than the writer is capable or called on to enter, there can be given no general solution; medical science is too far in arrear of the physical sciences to admit, like them, of the enunciation of its practically useful truths in terms at once concise and logically true. It resembles moral and political science in the complexity of its inquiries; the number and variety, and in many instances contingent importance, of its necessary data, bear the like proportion to its facts and ascertainable desiderata. In medical philosophy, in short, the neat and commodious aphorism is, if disencumbered of the voluminous postscript or commentary, too often worse than useless. In several of the above supposed, an acute and diligent physician might, after minute inquiry, be enabled approximatively to account for the occurrence actually observed in each, rather than any other; but an approximation is much less than science requires.

*Circumstances most favorable to Cold as a Morbific Agent.*

The circumstances which most favor the mischievous power of cold, and which I think it proper to specify here, are the following three:

1. *Exhaustion* by labour, or by stimuli (such as external heat), or by venery, or inebrity, or fasting.

2. *Internal fluxion,* unaccompanied by general excitement, as those of catarrh, digestion, catharsis, &c.

3. *General delicacy,* including convalescence, infancy, and age.

Exhaustion by labour or exercise is a state that gives to cold terrific power. Alexander the Great, it is notorious, narrowly escaped death from having taken the cold bath under such circumstances. An accident of a similar nature, but more unhappy result, lately occurred in the person of the son of the late premier of England. "Captain Canning," we are informed by the Rev. Dr. Walsh, in his Notices of Brazil, "had heated himself by violent exercise at rackets, and when he proceeded to Mr. Gordon's house, (in the vicinity of Funchal, in Madeira,) he entered the room he used to occupy, and having put on his morning-gown, he went down to a large tank in the grounds, where he undressed himself, and, plunging in, sank never more to rise with life." Such accidents are not very rare, but the most frequent occasion of death from cold in circumstances of exhaustion from exercise is a cold drink. This is a frequent cause of illness and death in hot seasons; examples of it are common even in our mild climate. The causation of sudden death from cold drink is obscure. Laudanum is the great remedy. Accidents of this nature might generally be avoided by taking drink in very small quantities and at intervals, instead of considerable draughts at once.

But sudden vicissitudes of temperature are, in the popular estimation, the great causes of disease; and, in their anxiety to shun the peril of one extreme, the vulgar, from ignorance of the true principle, often run into the other: thus it is universally held dangerous to go into the cold bath warm, although that state of organic energy of which cutaneous heat is an index is an indispensable condition of safety and advantage from cold bathing. The truth is, that danger arises not from warmth or even moisture of the surface, to the bather, but depends on the proportion borne by his reactive and calorific energies previously to bathing, to the frigorific power of the cold medium. There is abundant proof that even proper sweating by no means indicates unusual susceptibility of morbid effects from external cold. The impunity with which the Russ rolls in the snow, or plunges into the river, while reeking from his vapour-bath, is well known. The like practice prevails amongst some of the North Americans. I have myself had cold water thrown over me after having spent half an hour in a vapour bath at 115°, (and I am convinced that, had the vapour been much higher, the affusion would have been still less dangerous, much farther within the bounds of safety,) without any result except a lively sensation of cold, owing to the moderate temperature of the bath, and consequent slight excitement of the skin. Had the bath been much hotter, I should have been, I imagine, less sensible of the difference. The experiments of Dr. Fordyce, Sir C. Blagden, &c. (*Phil. Trans.* vol. 65,) plainly shew that, abstractedly from exhaustion and defective reactive calorific energy, there is no danger in exposure to cool air, even in a state of profuse perspiration. "During the whole day," says Blagden, "we passed out of the heated room (temperature 240°, 60°,) after every experiment, immediately into the cold air, without any precaution; after exposing our naked bodies to the heat, and sweating most violently, we instantly went into a cold room, and staid there even some minutes before we began to dress; yet no one received the least injury." The same impunity attended the passage into the cold air of Dr. Dobson and other experimenters at Liverpool. Internal reactive calorific power is in such cases unquestionably the great desideratum of protection.

The following passage from Scoresby is interesting in more than one point of view: "Where no sensible perspiration prevails," (he speaks of arctic regions, where great and often exhausting labour only could produce sensible perspiration,) "I have never seen in a healthy person any ill effect resulting from the greatest transitions. For my own part, indeed, whenever I have occasion to expose myself to a severe cold, I like to get the body well warmed, finding that, the more I am heated, the longer I can resist cold without inconvenience. Internal warmth, however, is greatly preferable to superficial heat, and the warmth produced by simple fluids, such as tea, soup, preferable to that occasioned by spirits. After the liberal use of tea I have often sustained a cold of ten degrees at the mast head for several hours without uneasiness; and, though I have often gone from the breakfast-room, where the temperature was fifty or sixty degrees, to the mast head, where it was ten degrees, and without any additional clothing except a cap, I never received any injury, and seldom much inconvenience, from the uncommon transition."

The truth seems to be that a vigorous adult has nothing to fear from any frigorific exposure of an ordinary kind, provided his organic power of evolving caloric be not exhausted or materially diminished by previous exertion. The increased sensibility to cold, and susceptibility of injury from that morbific agent, produced by the exhausting excitement (of the nervous, vascular, calorific, and possibly sanguineous functions,) caused by drunkenness, laborious exertion, venery, violent emotions, &c. strongly corroborate that opinion. Long exposure to moderate external heat often greatly increases susceptibility. Dr. Walsh mentions an instance in which, after long exposure to a temperature of seventy-two degrees, the occurrences of a seabreeze of sixty-one degrees so chilled every one as to force them to put on additional clothing. They had been going at the rate of 210 miles in twenty-four hours, in a temperature of seventy-three degrees in the sun, but just as they had passed Cape Frio (on the coast of Brazil,) "a strong breeze set in from the sea, which swept them along there at the rate of thirteen miles an hour," (one third faster than before.) "During the continuance of this breeze, the thermometer fell to sixty-one degrees, and the sense of cold, from the sudden transition of temperature, was quite painful. After bearing it for some time shivering on deck, it became intolerable, and we all went below, put on our warm clothing and dreadnoughts, and again appeared with thick woollen jackets and trowsers, as if we were entering Baffin's Bay, and not a harbour under one of the tropics."

I am convinced that diseases of cold are often caused by slighter changes than that recorded by Walsh. In persons whose occupations are indoor and sedentary, who live in warm apartments, pass their day in indolence, and use little locomotive exercise, there is often such susceptibility as to make them liable to disease from exposure even to the gentle and almost tepid or warm current caused even in close rooms by the draught through the chimney. Reaumur assures us, that a sudden change of temperature of four degrees is always sensible; and Macquart affirms, that a change of ten degrees is always disagreeably so: they both speak of healthy subjects only. Now Ritter has found, by experiment, that, in a close apartment, the air near the window differs three or four degrees from that of the middle of the room; the transition of temperature, therefore, to which a delicate person may be exposed on a change of seat from the immediate vicinity of the fire to that of a closed window, may amount to a dozen or even a score of degrees. But the importance of avoiding sudden transitions to persons whose reactive calorific energy is habitually feeble, and whose nerves have not been hardened by fresh blasts, &c. is now, I trust, sufficiently obvious. Nothing need be added about the inconvenience of exposure to cold after getting out of bed, &c.

The existence of *an internal fluxion unaccompanied by general febrile excitement*, is favorable to the morbific action of cold on those who are destitute of great reactive vigour: thus persons affected with chronic catarrh, chronic inflammation of the abdominal viscera, and other tedious phlogistic cachexies, bear cold very ill. During the process of digestion, exposure to cold is, cæteris paribus, more dangerous than at other times. The incautious use of the cold bath shortly after eating has often been attended by formidable results: the same holds of catharsis.

A third combination of circumstances favorable to the morbific action of cold is that *general delicacy characteristic of convalescence, infancy, and age.* It is a matter of daily experience that old people and convalescents suffer great inconvenience, and often fatal injury, from blasts or other atmospherical changes that would be quite innocuous to the healthy individual. It seems probable that the aged and sickly are, in many instances, to a greater and less extent in the same circumstances as inferior and young animals with regard to their calorific powers. It has not been experimentally proved, but yet seems likely, that the calorific function suffers in age and disease a diminution of power, so that the state of the old and infirm resembles more or less that of young birds, fishes, and other immature or inferior animals, whose temperature has been ascertained to vary with the medium by which they are surrounded. Even in healthy adults animal heat is by no means a fixed quantity. Although ninety-eight degrees be perhaps truly enough represented as the heat of man, it is still but the average: individuals exceed or fall short of that degree according to age and other circumstances. From Davy's experiments, it would seem that that average is too low for certain foreign countries, and for whole extra-European races of men. In practical writers there are numerous examples of animal heat depressed, for considerable periods of time, below the standard of health. The capability of resisting external cold by the compensative property of the calorific function is, for these and other reasons, I suspect, often degraded in age and infirmity. Tender infants also, I think it highly probable, have not yet attained to the full vigour of adult calorific power, and are therefore victims, on a very large scale,* to

* "Prenez mille enfants a leur naissance," says a learned writer, now bethe morbific energies of cold from an organic debility, similar to that which lays age and infirmity open to its attacks.

*Forms of Cold most dangerous.*

The principal and most active forms of morbific cold met with in practical life are three: *moist atmospheres, damp clothing*, and *currents of air*.

Moisture is not of itself injurious to health. Moist warm atmospheres are indifferent to the vigorous, and they are generally favorable to the weakly. *Wet* summers are healthful in this country, provided they are not cold; the summer of 1797 furnishes a very striking proof of this truth. "From the middle of May it was," says HEBERDEN, "one of the wettest ever remembered, it was nevertheless in every respect a healthy year." Not so, however, *wet cold* seasons. BATEMAN assures us "that the succession of rains to heat" (i. e. of a cool or cold moisture to warmth,) "is amongst the most active causes of disease of the chest and abdomen," which are the most destructive complaints in this metropolis. "A foggy atmosphere," he again observes, "acts much more injuriously than a clear (i. e. comparatively dry) one of equal cold. Indeed, there is," he assures us, "no condition of the air so invariably pernicious, so chilling and oppressive to the organs of respiration, as that frequent combination of frost with fog in the metropolis." Of the truth of the preceding observations of that judicious physician (Bateman,) I have had frequent experience amongst the poor inhabitants of Westminster for the last three years, during which I have had considerable opportunities of watching the operation of weather and season.

The danger of inhabiting or sleeping in *damp apartments*, is proved by examples of daily occurrence in private life. The superior morbific activity of a *damp atmosphere* depends on its superior conductive power. A humid air absorbs free caloric with much greater avidity and rapidity than a dry.

*Damp clothing* is another active and dangerous form of cold. The mischievous energy of wet clothes is so well known, as to require no illustration. The great capacity of evaporating water for the matter of heat, is the cause. The frigorific power of damp clothing may be conceived from this consideration: that the only protection or antagonist influence that man requires to enable him to defy the summer fires of fore me, "a peine ont ils va le jour qu'il en perit vingt-trois; la dentition en emporte cinquante; les convulsions, les vers, les coliques du premier age, enlevent plus du quart du deux cent soixant-dix-sept; la moitié de ce nombre de morts est due sur-tout a l'air froid et humide dans les campagnes," &c.

Sahara or South Carolina, is the power of cutaneous exhalation. Although inhaling and immersed for some time in an atmosphere exceeding very far the temperature of boiling water, the bakers' girls were found, by Reaumur, to have pertinaciously retained their normal heat. After twelve or fifteen minutes' immersion in an atmosphere many degrees above 212°, Blagden, Bankes, Dobson, and other experimenters, found their thermometrical heat little differing from that of ordinary health. Such is the frigorific power of perspiration, or, in other words, of evaporation from the surface.

But *currents of air*, perhaps, of all causes of diseases of cold, are the most active and extensively mischievous. *Damp clothes* may be avoided; *foggy atmospheres*, and extremely *humid cold winds*, are unknown in many seasons and climates: but *currents of air* must be encountered. The atmosphere is constantly in a state of agitation; its intestine and progressive motions, while, on the one hand, they promote our well-being by ventilation, endanger, on the other hand, our health and our existence by their refrigerant operation. The destructive power of exposure to cold winds without adequate protection, is strikingly illustrated by the narrative published by Dr. Currie, in the Philosophical Transactions for 1792. "Of several individuals that clung to the wreck, two sat on the only part that was not submerged: of the others all were constantly immersed in the sea, most up to the shoulders; three only perished, two of whom were generally out of the sea, but frequently overwhelmed by the surge, and at other times exposed to heavy showers of sleet and snow, and to a high and piercing wind." Of these two, one died, after four hours' exposure; the second died three hours later, "although a strong healthy man of twenty-eight, a native of Scotland, in the flower of life, early inured to cold and hardship, and very vigorous both in mind and body." The third that perished had been a weakly man. The remaining eleven who had been more or less completely *submerged*, were taken from the wreck next day, after twenty-three hours' exposure, and recovered. The person amongst the whole who seemed to have suffered least was a negro: of the other survivors, several were by no means strong men, most of them had been inured to the warm climate of Carolina." In the case of the two first that perished the morbific power of the "high piercing wind" was aided no doubt very powerfully by evaporation. In Dr. Currie's account of his experiments on the cold bath, we have the following interesting illustration of the superior refrigerant power of wind or air in motion. After continuing in the water fifteen minutes, the subject of some of his trials exhibited

"little or no diminution of his heat in rising into the air in a perfect calm, though during a frost; while the like exposure in a second trial, under similar circumstances, but with a north-east wind blowing sharply, produced a rapid diminution (of animal heat,) though the air was many degrees warmer" than in the preceding experiment.

I have above cited several examples of even death instantaneously produced by the chilling influence of a piercing north wind. Every valetudinarian is aware of the inconvenience and even danger of exposure to blasts from chinks and other apertures in rooms otherwise close.

### *Prevention of Diseases of Cold.*

The remarks I have to make in this section will come under the head of *Clothing*, *Exercise*, *Internal Heat*, or stimulating ingesta, and *Diaphoretic Means*, as hot diluents, bed-heat, &c.

Every considerable augmentation of refrigerant influence requires, on the part of the subject exposed, proportionate precautionary means for the protection of health: these preventive measures must consist either of increased clothing or of the use of means capable of compensating for defect of personal coverings, by diminution of intrinsic organic susceptibility. The class of preventive means last alluded to will be by and by considered, under the heads of exercise and stimulating ingesta: at present I shall confine myself to the question of clothing.

Transition from a tranquil into an agitated or progressive atmosphere, as from indoors into the open air, from the inside of a stage-coach to the outside, &c., is accompanied by a great increase of the refrigerant power, which the frame has to encounter, and will, in many instances, above all if moisture be present, require additional protective covering. When the exposure is but short, or the weather is fine, or the constitution vigorous, and reactive energy therefore ample, such precaution, no doubt, will generally be quite unnecessary: yet those compensative conditions must be often wanting in a greater or less degree; and exposure, therefore, not provided against by appropriate internal or external means, will often prove hazardous, and sometimes fatal. In how many cases has phthisis been traced to an indiscretion of the sort now alluded to; to a journey on the top of a stage-coach in bad weather, or by night with insufficient clothing, &c. In how many instances have youth, and accomplishment, and loveliness, fallen victims to the noxious influence of cool, perhaps damp, out-of-door atmospheres, in passing from one rout to another, or in returning from scenes of splendid riot to domestic solitude and repose.

In passing from a state of activity or exertion to one of relative quietude, precautions are often required for security: such transitions occur when horse or foot exercise is exchanged for riding in an open carriage, or gestation on the water, and obviously demand the like precautions with transitions from walking, running, &c., to sitting, lying down, &c. But of all conditions that require provident measures, that of sleep stands most in need of them. In that condition the calorific function is less excited, less exposed to incidental stimulation from physical agents, or moral impulses, or muscular exertion, than in any other. Less heat is evolved; the body is much more readily chilled, the cutaneous functions more easily disturbed, and every derangement of internal parts, producible by frigorific impressions on the skin, is more promptly effected. In the state of sleep, it is therefore, if ever, necessary to guard against exposure to cool moisture, currents of cool air, and every other cause of diseases of cold. All this is very plain, and is generally known, and requires no further notice. Before quitting this topic, however, I would briefly enter my protest against the absurd and mischievous extreme to which many, perhaps most people, carry the use of woollen and other night clothing. It is common for females, in particular, who seldom, amongst the richer classes at least, know the comfort, the real luxury, of woollen or chamois coverings for the shoulders, chest, feet, &c., and who wear below the knee, on the arms, upper part of the chest, neck, or head, either slight or no covering, to retire to sleep on beds of feathers, under half a dozen or more folds of one material or another, mostly woollen, and this in soft, nay even in summer, weather, and with every avenue for fresh air, every door and window, closed, and bedcurtains perhaps drawn closely all around: from such violent transitions what wonder if inconvenience result! The sleep is more or less disturbed by dreams and feverish uneasiness; the strength is not properly recruited, and the sleeper awakes unnerved, languid, indolent, often hot or chilly, generally anorectic. Under such circumstances, a susceptibility to inconvenience and injury from cold, above the average, may reasonably be looked for, and will, I believe, seldom fail, if occasion offer, to shew itself. Nor is the relaxation attending long immersion in warm air the only disadvantage in such cases; for there is obviously the further one of long-continued respiration of an impure atmosphere to be taken into the account: a disadvantage of no trifling importance in the cases of such as retire early to small rooms, and emerge into daylight after protracted slumbers.

Another point in which many fail, is the adaptation of clothing to season, weather, &c. No one questions the propriety of such adaptation in the abstract; but the number of those that commit the grossest errors on this subject in practice is enormous. What can be more obvious than the temerity of wearing the same kind and quantity of clothing in the heats of summer and frosts of winter: yet there are not wanting in the very first rank of the medical profession persons chargeable with such imprudence. I recollect very well the substance of an argument I once had with a fellow-traveller, an Austrian cadet, on his way through mountains, in mid-winter, *en voiture,* from Vienna to Laybach. He obviously suffered inconvenience from want of warmer clothing, yet would not admit the propriety of adding even a flannel vest to his wardrobe. He considered it, he told me, "militärisch," soldier-like, to dispense with woollen under-coverings. A like answer would no doubt be given by many defaulters on this side of the water. Ladies would hold it to be *feminine,* gentlemen, *manly,* &c., to dispense with the extra under-clothing proper for winter and cold weather. But indolence, temerity, and fine breeding, are bad protectives against inclement seasons.

*Exercise.* An observant individual can seldom fail to know when, from universal weakness or incidental exposure, he is in danger from external cold; and a provident man will easily, in general, foresee future exposure. When actually exposed, the great prophylactic is muscular exertion, and, if possible, locomotive exercise. Ritter's advice is excellent, when he recommends that we should counteract the chilling influence of a draught or of a damp atmosphere, to which we are constrained to expose ourselves, by proportionably increased exercise, in order that we may be enabled to compensate for the augmented expenditure of caloric by an increased evolution of it. The calorific power of general muscular exertion is such that, but for the antagonist frigorific power of cutaneous exhalation and vaporization, there can be no doubt that even moderate exercise would be incompatible with health, and that violent locomotive exertion would, in comparatively tranquil atmospheres at least, prove destructive of life. It is so great, that, duly persevered in, and aided by clothing sufficient to protect the skin and extremities from the immediate contact of an intensely cold air, it has been, on innumerable occasions, found sufficient to bear man harmless through the most formidable trials, as the narratives of Parry, Franklin, Scoresby, and many others, abundantly testify.

Respecting the use of *hot drinks* and *aliments at once nutritive and stimulant*, before and during exposure, little need be said. All experience is in their favor; every traveller on our stagecoaches knows the protecting power of warm tea and coffee, punch, &c.; there is even unequivocal experimental proof of the power of stimulant drinks to sustain the animal temperature under exposure. During my experiments on the cold bath, I found, in some trials with warm drinks and wine, (taken before immersion,) the sensation of cold little less lively indeed, and the access of shivering little retarded; but the pulse and heat under the tongue were much less reduced by the cold than in other trials made without such preparation. As a preparative, however, for protracted exposure to cold, &c., pure vinous liquors are obviously unsuitable means: the excitement they produce is transitory, and is followed by dangerous depression of calorific power: and their repeated and free use is, amongst other objections, liable to this, that it favors that somnolency which is one of the most perilous effects of cold. I have little doubt that the protective power of punch, negus, &c. is more owing to the hot water than to the pungent spirit.

The fourth division comprises the means of cutting short incipient diseases of cold. On the supervention of chilliness and other symptoms, effects of recent exposure to cold, such as slight headach, horripilation, dejection of spirits, hoarseness, slight sorethroat, coryza, lachrymation, cold feet, anorexia, lumbar pains, &c., we should have immediate recourse to the shelter of a warm bed; all solid aliment should be withheld; our only ingesta should be warm diaphoretic drinks. Diluted vinous liquors taken warm, such as weak hot punch or negus, are often excellent diaphoretics in such cases. But, in general, the alcoholic ingredients may be safely dispensed with, and when the excitement is considerable and headach is present, it cannot, without rashness, be recommended. The preceding measures are usually sufficient, if early enough employed, to cut short incipient derangements from cold. Where irritation is considerable, which is indicated by flying pains in the back and limbs, lively sense of cold, smart shivering, &c., opiates had better be employed in addition to the means already mentioned: for this purpose Ritter highly extols a combination of opium and camphor, two or four grains of the latter with from the eighth to a fourth part of a grain of the former every second hour, until the horrors, headach, pains, &c. shall have vanished or greatly declined. I have no doubt of the utility of such a combination; but pure laudanum or opium combined with warm diluents will probably be found fully as efficient. Dover's powder is also an excellent remedy. Another remedy, at once efficient and agreeable, is the common effervescing draught, containing half a scruple of nitre, a drachm (more or less) of the compound tincture of camphor, and in some cases half a drachm or more of nitrous æther, and as much of ipecacuanha wine, to be repeated every third, fourth, or sixth hour. Where the feeling of cold, as evidenced by horripilation, rigors, &c. is lively, warm bathing, local or general, followed up by some of the remedies just proposed, is very proper.

Prevention of disease is better than cure: it implies a more masterly degree of skill and power in the prescriber, and a smaller expense of care and vital power on the part of the sick. In practical medicine, the first indication, in the dignity as well as time, is prevention: in other words, the avoidance or counteraction, as far as possible, of morbific agencies; and when illness arrives, the employment, without loss of time, of the means best calculated to disperse the earlier groups of organic preternatural conditions or symptoms, and thus, by anticipation, get rid of the complications and difficulties so soon superinduced and accumulated upon primary simple and tractable derangements by the influence of sympathy and habit: with these views, I have thought it advisable to append to my observations on the morbid effects of cold, remarks on the circumstances that most favor the action of morbific cold, on the means best calculated to neutralize its agency, and on the remedies that should be employed after exposure to prevent the establishment of any injurious effect or regular disease of cold: on the plan, as on the execution, it is the reader's province to decide.

